# Non-Communicable Diseases in Sub-Saharan Africa: The Case for Cohort Studies

**DOI:** 10.1371/journal.pmed.1000244

**Published:** 2010-05-11

**Authors:** Michelle D. Holmes, Shona Dalal, Jimmy Volmink, Clement A. Adebamowo, Marina Njelekela, Wafaie W. Fawzi, Walter C. Willett, Hans-Olov Adami

**Affiliations:** 1 Channing Laboratory, Department of Medicine, Brigham and Women's Hospital and Harvard Medical School, Boston, Massachusetts, United States of America; 2 Department of Epidemiology, Harvard School of Public Health, Boston, Massachusetts, United States of America; 3 Faculty of Health Sciences, University of Stellenbosch, Cape Town, South Africa; 4 Department of Surgery, College of Medicine, University of Ibadan, University College Hospital, Ibadan, Oyo State, Nigeria; 5 Department of Physiology, Muhimbili University of Health and Allied Sciences, Dar es Salaam, Tanzania; 6 Department of Nutrition, Harvard School of Public Health, Boston, Massachusetts, United States of America; 7 Department of Medical Epidemiology and Biostatistics, Karolinska Intitutet, Stockholm, Sweden

## Abstract

Michelle Holmes and colleagues argue that there is an urgent need for longitudinal cohorts based in sub-Saharan Africa to address the growing burden of noncommunicable diseases in the region.

## Introduction

We believe there is an urgent need for longitudinal cohorts based in sub-Saharan Africa (SSA). This conclusion is drawn from the fact that non-communicable diseases (NCDs) cause a large and growing disease burden (please see Box 1) [1]–[6].

**Box 1. Selected Statistics on Non-Communicable Disease Burden in Sub-Saharan Africa**

**Non-communicable disease (NCD) burden**

- Over 80% of deaths from non-communicable diseases worldwide are estimated to occur in low- and middle-income countries [2].

**Cardiac diseases**

- The World Health Organization projects that the number of deaths from ischaemic heart disease in the African region will double by 2030 [3].

**Stroke**

- In 2004 stroke was estimated to cause 3% of all deaths in Africa and 52% of vascular deaths [4].

**Diabetes Mellitus**

- The prevalence of diabetes mellitus in Africa is predicted to increase by 80% in 20 y [5].

**Cancer**

- One in five deaths from NCDs in adults over 45 y in Africa is estimated to be caused by cancer [6].

In the past, public health in SSA has focused on communicable diseases. The advent of HIV/AIDS reinforced this image of infections as SSA's major health burden. However, NCDs, including cardiovascular diseases, mental illnesses, trauma, cancer, and diabetes, are now major sources of morbidity and mortality and are projected to overtake infectious diseases by 2030 [7],[8]. We argue that SSA lacks adequate resources to respond to this problem.

Prospective cohort studies can be used to study multiple complex diseases and risk factors simultaneously over an individual's lifetime. Such studies have proved crucial in understanding the etiology, course, and outcome of NCDs in other populations and have informed the design of prevention programs. In addition, cohort studies provide an incomparable resource for the training of public health researchers. Because the payoff from cohort studies continues—and often grows—over time, they are a long-term investment in public health.

In order to highlight the potential impact of cohort studies in SSA, we compared published literature on NCDs from longitudinal studies in high-income countries to publications from Africa. Further, we estimated the costs of establishing cohort studies in SSA and describe the response needed to correct the disparities in research investment between SSA and the world's more wealthy regions.

## What We Have Learned from Prospective Cohorts in the Wealthy Countries

In 2001, Pettiti prepared a list of 55 important findings from epidemiological studies for the Epidemiology Monitor [9]. This list, reproduced and recently revised by Dr. Pettiti (personal communication, D. Pettiti) in Table 1, represents only a partial inventory of epidemiological discoveries and does not refer exclusively to cohorts but all epidemiological designs.

**Table 1:** Epidemiology triumphs.

Category	Disease	Risk Factor	Direction
**Alcohol**	Esophageal cancer	Alcohol interaction with smoking	IR
**Viruses**	Liver cancer	Hepatitis B virus	IR
	Burkitt's lymphoma	Epstein Barr virus	IR
	Kaposi's sarcoma	Herpes simplex virus type 8	IR
	Cervical cancer	Human papilloma virus	IR
	Nasopharyngeal cancer	Epstein Barr virus	IR
	Yellow fever	Transmitted by mosquitoes	IR
	Creutzfeldt-Jacob disease	Prions (interaction with genotype)	IR
**Bacteria**	Cholera	“Something in water” (vibrio cholera)	IR
	Peptic ulcer	Helicobacter pylori	IR
	Puerperal fever	“Something on doctors' hands” (group B Streptococcus)	IR
**Nutrition**	Pellagra	“Something in food” (niacin)	P
	Neural tube defects	Folic acid, folate	P
	Oral clefts	Folic acid, folate	P
**Occupation**	Lung cancer	Asbestos (interaction with smoking)	IR
	Lung cancer	“Something in uranium mines” (interaction with smoking)	IR
	Bladder cancer	Aniline dye	IR
	Mesothelioma	Asbestos	IR
	Angiosarcoma	Vinyl chloride	IR
	Nasal cancer	Nickel	IR
	Male infertility	Dibromochlorpropane (DBCP soil fumigant)	IR
	Scrotal cancer	Chimneysweep	IR
**Environment**	Cancer	Arsenic	IR
	Chloracne	Dioxin	IR
	Polychlorinated biphenyls	Hyperpigmentation at birth	IR
	Chlordecone (Kepone pesticide)	Tremor	IR
	Methyl mercury exposure from eating fish due to water contamination during manufacture of plastics	Severe neurologic disease/cerebral palsy with intrauterine exposure. Sensory disorders in adults	IR
	Dental caries	Fluoride	IR
**Drugs & devices**	Myocardial infarction	Aspirin	P
	Microagnathia	Iso-retinone during pregnancy	IR
	Pelvic inflammatory disease	Dalkon shield IUD	IR
	Septic abortion	Dalkon shield IUD	IR
**Hormones**	Clear cell adenocarcinoma of the vagina	Diethylstibestrol	IR
	Venous thromboembolism	Oral contraceptives	IR
	Venous thromboembolism	Post menopausal estrogen	IR
	Ovarian cancer	Oral contraceptives	P
	Endometrial cancer	Oral contraceptives	P
	Endometrial cancer	Post menopausal estrogen	IR
	Iron deficiency anemia	Oral contraceptives	P
	Benign breast disease	Oral contraceptives	P
	Myocardial infarction	Oral contraceptives (interaction with smoking)	IR
	Ischemic stroke	Oral contraceptives (interaction with hypertension; modified by dose)	IR
**Genetics**	Breast cancer	BRCA1, BRCA2 mutations	IR
	Ovarian cancer	BRCA2 mutations	IR
	Colon cancer	APC1 mutations	IR
**Miscellaneous**	Toxic shock syndrome	Super absorbent tampons	IR
	Sudden infant death	Prone sleep position	IR
	Reye's syndrome	Aspirin (interaction with infection)	IR
**Smoking**	Lung cancer	Smoking	IR
	Coronary disease	Smoking	IR
	Hemorrhagic stroke	Smoking	IR
	Ischemic stroke	Smoking	IR
	Abdominal aortic aneurysm	Smoking	IR
	Peripheral vascular disease	Smoking	IR
	Parkinson's disease	Smoking	P
	Ulcerative colitis	Smoking	P
	Laryngeal cancer	Smoking	IR
	Intrauterine growth retardation	Smoking during pregnancy	IR
	Toxemia/preeclampsia	Smoking during pregnancy	P

From Petitti D, Epidemiology Monitor, July 30, 2001, and revised May 2009, http://www.epimonitor.net/EpiMonday/Triumph62501.htm, used with permission. IR, increased risk; P, protective.

Colditz and Winn recently proposed criteria by which the success of large cohort studies can be judged [10]. The criteria they suggest are: (1) discovery, (2) development, and (3) delivery. Discovery refers to the ability to explain disease etiology and is measured by numbers of publications and the impact factor of the journals in which they are published. Development refers to the provision of a basis for developing prevention and control measures for populations at risk. Development is measured by contribution to the determination of factors causing disease, providing scientific support for prevention, clinical guidelines and randomized trials, and quantification of the preventable burden of disease. Delivery refers to the implementation of findings from the discovery and development phase by clinicians, public health practitioners, policy makers, and the general public. Delivery is measured by health policies, industry applications, and public awareness. Although these criteria were applied to studies of cancer epidemiology, they are more broadly applicable. When applied to the Nurses' Health Study (NHS), a longitudinal cohort begun in 1976 with 121,700 US female registered nurses, this one study contributed importantly according to the Colditz and Winn's criteria. Because cohorts are able to focus on multiple outcomes, they can demonstrate the full public health impact of lifestyle factors.

## International Disparity in Investment in NCD Research

### International Disparity in Publications

We attempted to quantify numbers of publications in the following manner. We searched the PubMed on-line database [11] up to November 26, 2009. We included the Medical Subject Headings (MeSH) “cohort studies” in combination with “Africa South of the Sahara” and two NCDs: “stroke” and “diabetes mellitus.” Each condition was limited to being a major subject matter. We repeated each search, changing “Africa South of the Sahara” to “United States,” “China,” “India,” and “Europe.” We used the PubMed limit function to limit each search to those articles involving humans, having an abstract, and written in English. We counted citations retrieved by these strategies.

We estimated the number of people with diabetes and the number at risk for stroke (those with hypertension) in each area from the following sources. Total population was from The WHO's World Health Statistics 2008 [12]. The prevalence of hypertension in the US was from the National Center for Health Statistics [13]. The prevalence of hypertension in Africa [14]–[18], Europe [19], China [20], and India [21] was estimated from several sources. The prevalence of diabetes for all countries and regions was from the International Diabetes Federation 2009 Diabetes Atlas [5].

**Figure 1:**
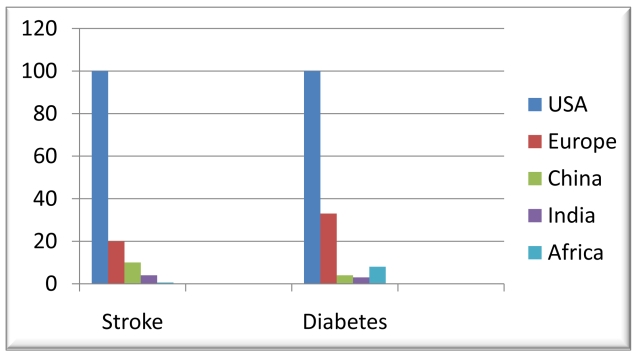
Ratio of numbers of publications from cohort studies by condition (graph). Ratio of numbers of publications from cohort studies by condition (stroke, diabetes mellitus) per million people with the disease (diabetes) or at risk of the disease (stroke) in Africa, Europe, China, India, and the US.

**Figure 2:**
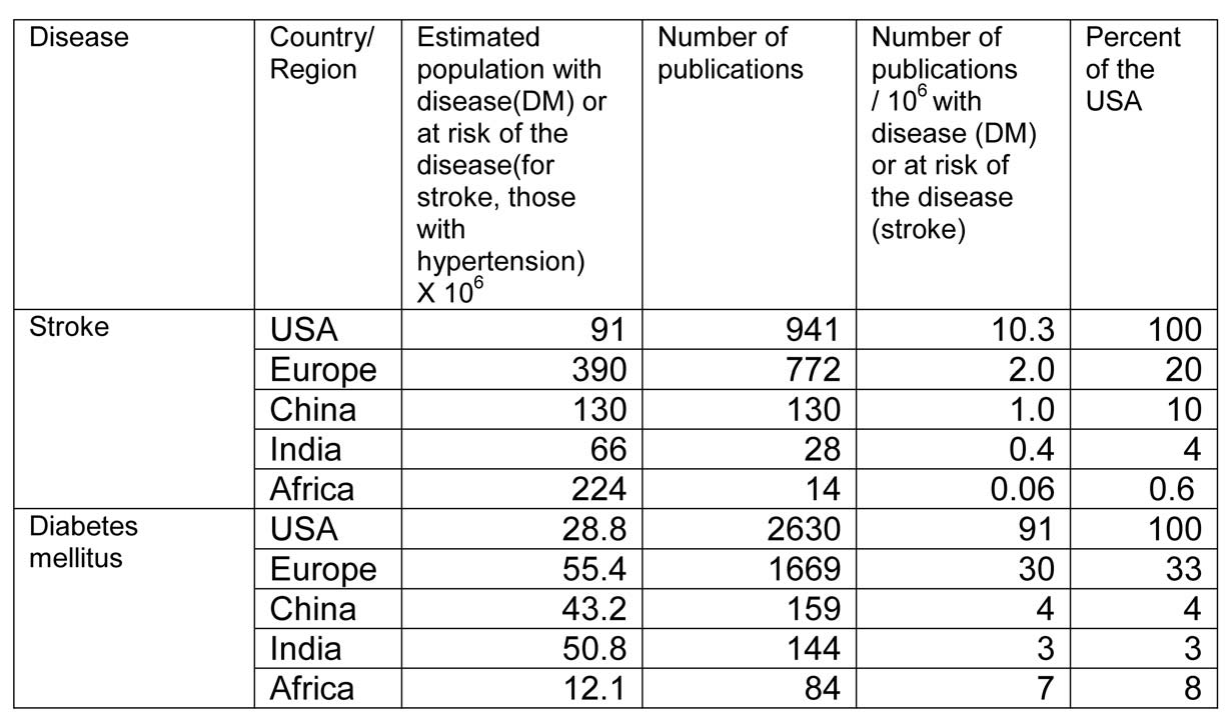
Ratio of numbers of publications from cohort studies by condition (table). Ratio of numbers of publications from cohort studies by condition (stroke, diabetes mellitus) per million people with the disease (diabetes) or at risk of the disease (stroke) in Africa, Europe, China, India, and the US, and ratio of these publications with US as the reference group.

We calculated the number of publications per million people for each condition in each geographic region (Figures 1 and 2). Continental Africa produced a tiny fraction of the publications from cohort studies per affected population compared to the US: 0.6% for stroke and 8% for diabetes.

### International Disparity in Adults Enrolled in Long-Term Cohorts

No central database of numbers of adults enrolled in long-term cohorts of NCDs exists. We have relied on our knowledge of the epidemiology field and that of our colleagues to estimate the proportions of populations enrolled. This method is admittedly crude and we have assuredly missed many. For instance, large populations in North America and Europe are monitored through data linkage to the health care system and are regularly used as cohorts. However, we believe that one brief example can provide a conservative estimate of the magnitude of the disparity.

In the US there are 18 cohorts and 2.9 million participants that are part of The Pooling Project [22].

For Africa we included the following cohorts. The Birth to Twenty Cohort studies the health of young adults born in 1990 in Johannesburg-Soweto, South Africa [23]. The Heart of Soweto is a health facility-based South African study that enrolled 4,162 cardiovascular disease cases in 2006. The Women's Health Study of Accra, Ghana enrolled 1,328 women to study the prevalence of communicable diseases and NCDs [24],[25]. The total cohort enrollment from these three sources is 8,763.

Using the populations of the US and Africa from the WHO's World Health Statistics 2008 [12], we found that compared to the US, Africa had 0.1% as many cohort enrollees. Given that there are certainly more US enrollees than we have counted, and unlikely to be large African cohorts that we have missed, the disparity is likely to be even larger. Recent calls to create even more US cohorts to study gene environment interactions [26],[27] would increase this disparity.

INDEPTH is an organization that evaluates population demography in developing countries, including 12 in SSA [28]. Because they assess primarily births, deaths, migration, and not risk factors or longitudinal data, we have not included them in our count of SSA cohorts. Although some have NCD projects, a massive input of resources would be required to convert them to true prospective cohorts.

### International Disparity in Research Money Spent

In 2008 the Global Forum for Health Research published the document “Monitoring Financial Flows for Health Research 2007” [29], the preface of which reads:

*Since [1990] the landscape of health research for development has changed in important ways:*

- *global expenditure on health research has more than quadrupled to over US$125 billion in 2003;*
- *there are many more actors engaged in funding or conducting health research relevant to the needs of developing countries;*
- *but the epidemiology of diseases has shifted substantially, so that many developing countries are now experiencing high burdens of non-communicable diseases such as cancer, diabetes, heart disease, and stroke, as well as continuing high burdens of infectious diseases and injuries.*

*As a result of these changes, the total global expenditure applied to research relevant to all the health problems of developing countries cannot be estimated with any meaningful degree of accuracy.*

Yet Chapter 5 of this same document, called “Using Bibliometrics to Inform Cancer Research and Spending,” used publication numbers and estimated cost per paper to estimate total world cancer research expenditures. This provides assurance that the disparity in publications calculated previously can provide insight into the disparity in money spent.

### International Disparity in Scientific Person-Power

Economic development is intrinsically linked to science and technology produced by academic, technical, and professional institutions [30]. Currently, many SSA countries have small scientist pools because of limited research funding and few viable career paths. Lasting, indigenous capacity building in public health requires the development of scientific training and capabilities for research.

## What Africa Can Teach Us

There is no reason to doubt that smoking, obesity, high salt intake, sedentary lifestyle and pollution will have adverse health effects in Africa as in other places.

Why then do we need to do cohort studies in Africa? We suggest the following reasons: to study population-specific disease burden; genetic heterogeneity; unparalleled geographic, social and cultural diversity; practices and secular trends that may be unique risk factors; and to stimulate political will for health promoting change.

Scientific findings from North America and Europe may not be applicable to other regions. Longitudinal studies track changes in risk factors over time and provide health planners with information on disease burden. One example would be hypertension prevalence. This has been considered a problem of more wealthy, sedentary and obese urban but not rural African populations [15]. Yet blood pressure measurements from cross-sectional studies in several rural African locations have found a hypertension prevalence of 11%–25%, similar to that in the cities and in high income countries [15],[16]. A cross-sectional study of 1,500 rural villagers over age 15 in three nations (Tanzania, Malawi, and Rwanda) found 23% had hypertension that was independent of obesity (mean body mass index was 21 kg/m^2^) and associated with diet [31]. Others have confirmed these findings [17],[18]. The assessment that hypertension is mainly a problem of cities is therefore wrong. Global dietary changes will affect the trajectory of hypertension prevalence in the future, and cohorts will give information on this trajectory that the cross-sectional studies cannot.

Diet is another location-specific cultural practice. Although we know from studies in high income countries that consumption of whole grain based on mostly wheat and rice is associated with lower risk of diabetes [32], this may not be applicable in East Africa where the staple is corn. We need to research local foods that confer protection to formulate interventions.

History is also location specific. With the exception of studies of starved Europeans during World War II [33], studies of the West's obesity epidemic have all been among populations with a lifetime of more than adequate energy intake. In Africa, under-nutrition remains common [34] yet coexists with the global obesity epidemic [16],[35],[36]. Evidence from high income countries shows that intrauterine growth retardation predisposes to increased risk of cardiovascular and metabolic diseases [37]–[41]. The effects of the collision of these two secular trends in Africa (recent under-nutrition and rapid introduction of obesity-enhancing lifestyle factors) cannot be fully anticipated.

The high prevalence of infectious diseases such as HIV in SSA along with NCDs is another location-specific opportunity. Inflammation common to infections is increasingly known to be important for cardiovascular, metabolic, and neoplastic disease [42]–[44]. SSA presents the opportunity to better understand this pathophysiology.

Adding to the complexity of locale-specific behavior and history is genetic diversity. Humans first evolved in East Africa, and thus African genetic diversity is greater than elsewhere [45]. Studying the interaction between environmental factors and genes elucidates disease mechanisms. If one multiplies the extended range of cultural practices and historical circumstances by the genetic diversity found in Africa, the opportunities for scientific discovery become enormous (S. Dalal, M.D. Holmes, R. Ramesar. Advancing public health genomics in Africa through prospective cohort studies. *Journal of Epidemiology and Community Health* [In press]).

Even if smoking and intake of salt and refined grains has been shown detrimental in higher income populations, political will for change may be greater among policy makers, clinicians, and the public if shown in their own population.

Although cohort methodology developed in North America and Europe, high income countries may also gain from knowledge derived from African studies. For example, the obesity epidemic in the West is well developed; we have yet to apply our knowledge to interventions having substantial impact [46].

## Estimated Cost of African Cohorts

We propose cohort studies of 50,000 to 100,000 people in three or four countries covering west, east, and southern Africa. The higher number would increase enrollment of Africans to 532 per million, still only 5% compared to the US.

These studies will include the collection and storage of biological specimens, repeated exposure measurements, and ongoing ascertainment of disease among participants. Disease ascertainment poses particular challenges in countries without strong health systems. It will be important to choose strategies that accommodate local circumstances.

We propose to include populations that are urban, rural, and include men, women, and children. We have four overarching goals: (1) to measure disease burden; (2) to study the etiology of NCDs, the distribution of risk factors, and attributable risks; (3) to provide short-term results by nesting randomized intervention trials within the cohorts; and (4) to stimulate academic development and collaboration.

Ideal study sites would be those where African colleagues have some history of cooperation with the population. Sites with established assessments or interventions may offer cost savings. However, such sites are not likely to easily accommodate the additional proposed layers of complexity, including multiple research designs, multinational collaborators, and managing new enrollees with ongoing study participants separately.

### Cohort Costs

We estimated costs for establishing cohorts in two ways. The first was by comparison to the mail-based NHS costs for April 1, 2005 to March 31, 2006 in Table S1 (personal communication, S. Hankinson). Personnel costs are lower in Africa than in the US. We used the Source of International Wage Comparisons compiled by the financial firm UBS to calculate wage differences (Table S2) [47]. Using this method, we estimated the cost of an African cohort of 100,000 participants to be $1.2 million per year, or $12/person/year (Table S3).

Costs could be higher without a reliable mail system; thus we estimated African cohort costs a second way. We applied the costs of a cohort of 775 patients followed for HIV medication compliance in rural Uganda (personal communication, D. Bangsberg). One year costs were $494,256, or $638/person/year.

### Randomized Trial Costs

We used information from a randomized controlled trial (RCT) of single versus multiple doses of multivitamins in addition to ART for women with HIV in Tanzania to estimate the cost of a RCT (personal communication, W. Fawzi). The cost was $4 million for 5 y for 3,000 people. However, this included multiple laboratory measurements because participants were ill. Assuming half the cost for an intervention in well people, we estimate RCT costs to be as low as $135/person/year. We applied a range of $135–$270/person/year to our estimates.

The prevalence of hypertension in Africa is similar to the US [15],[16]. The Dietary Approaches to Stop Hypertension (DASH) trial significantly lowered blood pressure among 459 hypertensive Americans [48]. An illustrative intervention would be to do an “African DASH” trial substituting culturally appropriate foods to lower blood pressure among 500 Africans lasting 2 y. We propose doing a total of five similar interventions addressing diseases of the epidemiologic transition over 10 y. The costs for 500 people×2 y×($135–$270)×5 similar type interventions = $675,000 to $1.35 million.

### Training Costs

We assumed the costs of educating African scholars using tuition and living expenses at Harvard School of Public Health [49],[50]. We propose other concomitant models for training including lecturers in residence at African universities, short courses, and distance learning. We assumed a range of costs depending on the degree program (master's or doctorate) and whether the student was single or had a family. The cost per student given in Table S4 ranges from $110,000 to $269,000.

### Total Cost Scenarios

Table S5 summarizes the assumptions of the range of costs explained in this section. We assumed cohorts would extend at least 10 y; in a recent review successful large cohorts experienced exponential publication growth after 10 y [10]. We then applied high and low costs for the cohort, interventions, and for training, and combinations thereof in Table 2. The low estimate including interventions and training is $23.7 million for a cohort of 50,000 people in three countries each. The high estimate is for $2.56 billion for a cohort of 100,000 people in four countries each.

**Table 2:** African Cohort Initiative named PaCT (Partnership for Cohort Research and Training) – Estimation of range of costs for two scenarios.

	Four Countries		Three Countries	
	(*N* = 100,000/Country)		(*N* = 50,000/Country)	
	High	Low	High	Low
**Cohort**	2,000,000,000	48,000,000	750,000,000	18,000,000
**Intervention**	1,350,000	675,000	1,350,000	675,000
**Training**	9,296,000	6,720,000	6,972,000	5,040,000
**Totals**				
Cohort+Interventions	2,001,350,000	48,675,000	751,350,000	18,675,000
Cohort+Training	2,009,296,000	54,720,000	756,972,000	23,040,000
Cohort+Interventions+Training	2,010,646,000	55,395,000	758,322,000	23,715,000

Comparisons are useful. PEPFAR cost $18.8 billion for 5 y only for HIV/AIDS [51]. The Bill and Melinda Gates Foundation will spend $3.9 billion in 2009 on various causes including global health [52]. Harvard University's fundraising for fiscal year 2006 was $600 million [53].

The UK Biobank, a cohort of 500,000 people with a baseline assessment only and 8 y of follow-up linked to a national health record, will cost $104 million [54]. The similar Swedish LifeGene cohort of 500,000 people and with baseline assessment and 8 y of follow-up will cost $112 million (personal communication H.-O. Adami). Translated into costs per person per year, the UK Biobank is $26 and LifeGene is $28. The high and low estimates of the African initiative gives a range from $14 to $644/person/year for the added value beyond the European cohorts of repeated exposure measurements, embedded interventions, and academic capacity building. Other scenarios of different size and intensity could be entertained. For instance, a large company that already conducts periodic health examinations of employees might be economically converted to a cohort. Also, funding need not necessarily come from a single source. However, some costs for an African cohort will be higher than a similar cohort in Europe or North America. A generous budget for travel will be needed to allow newly trained personnel to return to the host institution for further contact and exchange of ideas, but also to enable experienced researchers to visit the sites frequently.

## Sustainability

Yach defined the following obstacles impeding attention to NCDs in developing countries: policy makers lack evidence of disease burden, beliefs that NCDs affect only the wealthy and elderly, that they arise only from freely acquired risks, and that their control is expensive, ineffective, and a lower priority than infectious diseases [7].

These obstacles will need to be addressed if cohort studies of NCDs are to be sustainable. Health systems will have to be strengthened to absorb the disease burden uncovered by research. Training programs in public health disciplines are also necessary for long-term career development for the next generation of researchers.

We believe that starting large cohort studies in Africa is a 21st century design to serve public health in Africa. It combines surveillance, epidemiology, and clinical trials, and could yield information relevant to the NCD epidemic in wealthy countries. The funding for such an initiative will require political leadership, a long vision of the possibilities, and patience in awaiting results. We hope that the growing burden of NCDs and the unfulfilled need for research and action will spur funders of global health to include NCDs in Africa in their agenda.

## Supporting Information

Table S1 Nurses' Health Study costs for the year April 1, 2005 to March 31, 2006, for ∼100,000 participants.
(0.03 MB RTF)

Table S2 International wage comparisons.
(0.04 MB RTF)

Table S3 Estimated 1 y cost of an African cohort of 100,000 participants, using the Nurses' Health Study as a prototype.
(0.03 MB RTF)

Table S4 Cost of degree programs at Harvard School of Public Health.
(0.03 MB RTF)

Table S5 African Research Initiative – Assumptions.
(0.04 MB RTF)
