# Supplementary material for: Non-Communicable Diseases in Sub-Saharan Africa: The Case for Cohort Studies
Source: PLoS Med. 2010 May 11;7(5):e1000244. doi: 10.1371/journal.pmed.1000244 (PMC2867939; doi:10.1371/journal.pmed.1000244)
Supplement: Table S1 — Nurses' Health Study costs for the year April 1, 2005 to March 31, 2006, for ∼100,000 participants. (0.03 MB RTF) [file pmed.1000244.s001.rtf]

Table S1:  Nurses' Health Study Costs for the year April 1, 2005 to March 31, 2006, for ~100,000 participants
Type of Cost	Costs, in USD	% of total	
Personnel costs	$1,813,771	64	
Consultants, supplies, laboratory, contractual,
facilities, administration	1,028,907	36	
Total direct costs	2,842,768	100	
Annual cost per participant	28		
