# Supplementary material for: Non-Communicable Diseases in Sub-Saharan Africa: The Case for Cohort Studies
Source: PLoS Med. 2010 May 11;7(5):e1000244. doi: 10.1371/journal.pmed.1000244 (PMC2867939; doi:10.1371/journal.pmed.1000244)
Supplement: Table S2 — International wage comparisons. (0.04 MB RTF) [file pmed.1000244.s002.rtf]

Table S2:  International Wage Comparisons
Region	City	Wage, in USD	Regional Average	Ratio of average African to US Wage	
Africa	Johannesburg	$4.90	
$2.43	


12%	
	Lagos	1.30			
	Nairobi	1.10			
United States	Chicago	21.20	
20.50		
	New York	21.70			
	Los Angeles	18.60			
