# Supplementary material for: Non-Communicable Diseases in Sub-Saharan Africa: The Case for Cohort Studies
Source: PLoS Med. 2010 May 11;7(5):e1000244. doi: 10.1371/journal.pmed.1000244 (PMC2867939; doi:10.1371/journal.pmed.1000244)
Supplement: Table S3 — Estimated 1 y cost of an African cohort of 100,000 participants, using the Nurses' Health Study as a prototype. (0.03 MB RTF) [file pmed.1000244.s003.rtf]

Table S3:  Estimated one year cost of an African cohort of 100, 000 participants, using the Nurses' Health Study as a prototype
Type of Cost	Costs, in USD	% of total	
Personnel costs
(~12% of the NHS)	~$200,000	17	
Consultants, supplies, laboratory, contractual,
facilities, administration
(same as the NHS)	~1,000,000	83	
Total direct costs	~1,200,000	100	
Annual cost per participant	12		
