# Supplementary material for: Non-Communicable Diseases in Sub-Saharan Africa: The Case for Cohort Studies
Source: PLoS Med. 2010 May 11;7(5):e1000244. doi: 10.1371/journal.pmed.1000244 (PMC2867939; doi:10.1371/journal.pmed.1000244)
Supplement: Table S4 — Cost of degree programs at Harvard School of Public Health. (0.03 MB RTF) [file pmed.1000244.s004.rtf]

Table S4:  Cost of degree programs at Harvard School of Public Health
2 year Master student low cost
(single)	$110,000	
2 year Master student high cost
(+spouse+2children)	156,000	
5 year PhD student low cost
(single)	200,000	
5 year PhD student high cost
(+spouse+2children)	269,000	
