# Supplementary material for: Non-Communicable Diseases in Sub-Saharan Africa: The Case for Cohort Studies
Source: PLoS Med. 2010 May 11;7(5):e1000244. doi: 10.1371/journal.pmed.1000244 (PMC2867939; doi:10.1371/journal.pmed.1000244)
Supplement: Table S5 — African Research Initiative – Assumptions. (0.04 MB RTF) [file pmed.1000244.s005.rtf]

Table S5:  African Research Initiative – Assumptions
	Scenario 1	Scenario 2	
Number of  countries(sites)	4	3	
Number of  people/country for cohort	100000	50000	
Number of years cohort	10	10	
Number of people in dietary RCT to reduce blood pressure	500	500	
Number of years for each intervention study	2	2	
Number of intervention studies during 10 year period, total	5	5	
Cohort High cost/person/year	638	638	
Cohort Low Cost/person/year	12	12	
Interventions High Cost	270	270	
Interventions Low Cost	135	135	
